# Molecular basis for occlusion of the jeilongvirus receptor-binding site by the elongated C-terminus

**DOI:** 10.1128/mbio.01501-25

**Published:** 2025-11-25

**Authors:** Alice J. Stelfox, Airah Javorsky, Robert Stass, Geoff Sutton, Kamel El Omari, Thomas A. Bowden

**Affiliations:** 1 Division of Structural Biology, Centre for Human Genetics, University of Oxford 240364, Oxford, United Kingdom; 2 University Grenoble Alpes, CNRS, CEA, IBS 27015, Grenoble, France; 3 Diamond Light Source, Harwell Science and Innovation Campus 70597 , Harwell, United Kingdom; 4 Rutherford Appleton Laboratory, Research Complex at Harwell 838907, Didcot, United Kingdom; Case Western Reserve University School of Medicine, Cleveland, Ohio, USA

**Keywords:** paramyxovirus, *Jeilongvirus*, virus–host interactions, structure, glycoprotein, viral attachment

## Abstract

Paramyxoviral transmission between hosts may be, in part, attributed to the ability of the viral envelope-displayed receptor-binding protein (RBP) to bind to cell surface receptors of different host species. We sought to elucidate the architecture of the receptor-binding head region of the RBPs presented by jeilongviruses, a group of emerging and genetically unique paramyxoviruses belonging to the genus *Jeilongvirus,* family *Paramyxoviridae*. Structure determination of J and Beilong jeilongvirus RBPs reveals that the proteins exhibit a prototypical six-bladed β-propeller fold, present a binding site with residues associated with sialic acid recognition and hydrolysis, and bear a close structural relationship with sialic acid binding hemagglutinin-neuraminidase (HN)-type paramyxoviral RBPs. Additionally, unlike other paramyxoviruses, jeilongviruses encode an RBP with an unusually long C-terminal extension. In our dimeric Beilong virus RBP structure, we find that the C-terminal extension exchanges a hat-like domain with the central region of the β-propeller of the opposing protomer through domain-swapping. The hat-like domain occludes residues putatively associated with sialic acid binding and hydrolysis, providing a structural rationale for the absence of observed hemadsorption and neuraminidase activity. The insights gleaned from this analysis expand our appreciation of the structural palette available to the plastic paramyxoviral RBP and how their architectures may be adapted to regulate host-cell interactions at the cell surface.

**IMPORTANCE:**

The paramyxovirus receptor-binding protein (RBP) plays a primary role in determining cell and species tropism. Here, we study the RBPs of jeilongviruses, a group of paramyxoviruses that present a distinctive RBP that encodes an elongated C-terminal region. While the jeilongviral RBP structurally categorizes with paramyxoviral RBPs that interact with sialic acid during host-cell entry, the unusually long C-terminal domain was found to sterically occlude the associated binding site, suggesting that the molecule has developed strategies for autoinhibition of receptor interactions. These data expand our understanding of the architectural space occupied by paramyxoviral RBPs and the structural elaborations that may be incorporated into the paramyxovirus genome to modulate native functionality.

## INTRODUCTION

Like other paramyxoviruses, viruses within the recently established genus, *Jeilongvirus*, family *Paramyxoviridae*, encode a negative-sense, non-segmented RNA genome. J paramyxovirus (JPV) (1–3) and Beilong paramyxovirus (BeiV) (4–8) are founding members of a genus that currently includes thirty-two members (3, 9) and genetically divides into two clusters, reflecting the differential use of small mammals or bats and felines as putative host reservoirs (10). Viral surveillance and discovery efforts have revealed that this group of pathogens exhibits a near worldwide distribution. Furthermore, although other paramyxoviruses are of substantial threat to human health and animal husbandry, little is known about the potential threat that jeilongviruses pose due to a paucity of knowledge about their pathobiology, tropism characteristics, and inter-species transmission potential.

Like other paramyxoviruses, jeilongviruses display two major proteins on the viral envelope surface, a receptor-binding protein (RBP) and fusion (F) glycoprotein, which function in concert to facilitate viral entry into a host cell (11). The RBP is presented on the viral surface as a dimer-of-dimers (12, 13), with each protomer formed of an N-terminal intraviral (IV) region, transmembrane TM domain, α-helical stalk, and a C-terminal receptor-interacting six-bladed β-propeller receptor-binding head domain. Depending on the paramyxovirus, the membrane distal six-bladed β-propeller domain interacts with proteinaceous or glycan host-cell surface receptors during host-cell entry. While there remains much to be elucidated about the range of host-cell surface receptors utilized by paramyxoviruses, three types of RBP classes are currently recognized: hemagglutinin-neuraminidase (HN), hemagglutinin (H), and glycoprotein (G). The protein-binding H-type RBPs from morbilliviruses interact with nectin-4 and signaling lymphocytic activation molecule F1, and the G-type RBPs from henipaviruses differentially interact with ephrins. Conversely, viruses within the Respirovirus, Orthoavulavirus, Metaavulavirus, Paraavulavirus, and Orthorubulavirus genera present HN-type RBPs, which bind and hydrolyze sialic acid, a carbohydrate (11). Paramyxoviral HN-type RBPs retain the seven conserved sialidase residues (14) and a hexapeptide motif (15), which all surround the sialic acid binding pocket (11). Interestingly, the Jeilongvirus RBP retains the majority of the residues responsible for sialic acid binding and hydrolysis, which are highly conserved across HN-type RBPs (14, 15). However, investigations of JPV indicate that the virus lacks the expected neuraminidase activity associated with HN-mediated viral egress (1, 3).

**Fig 1:**
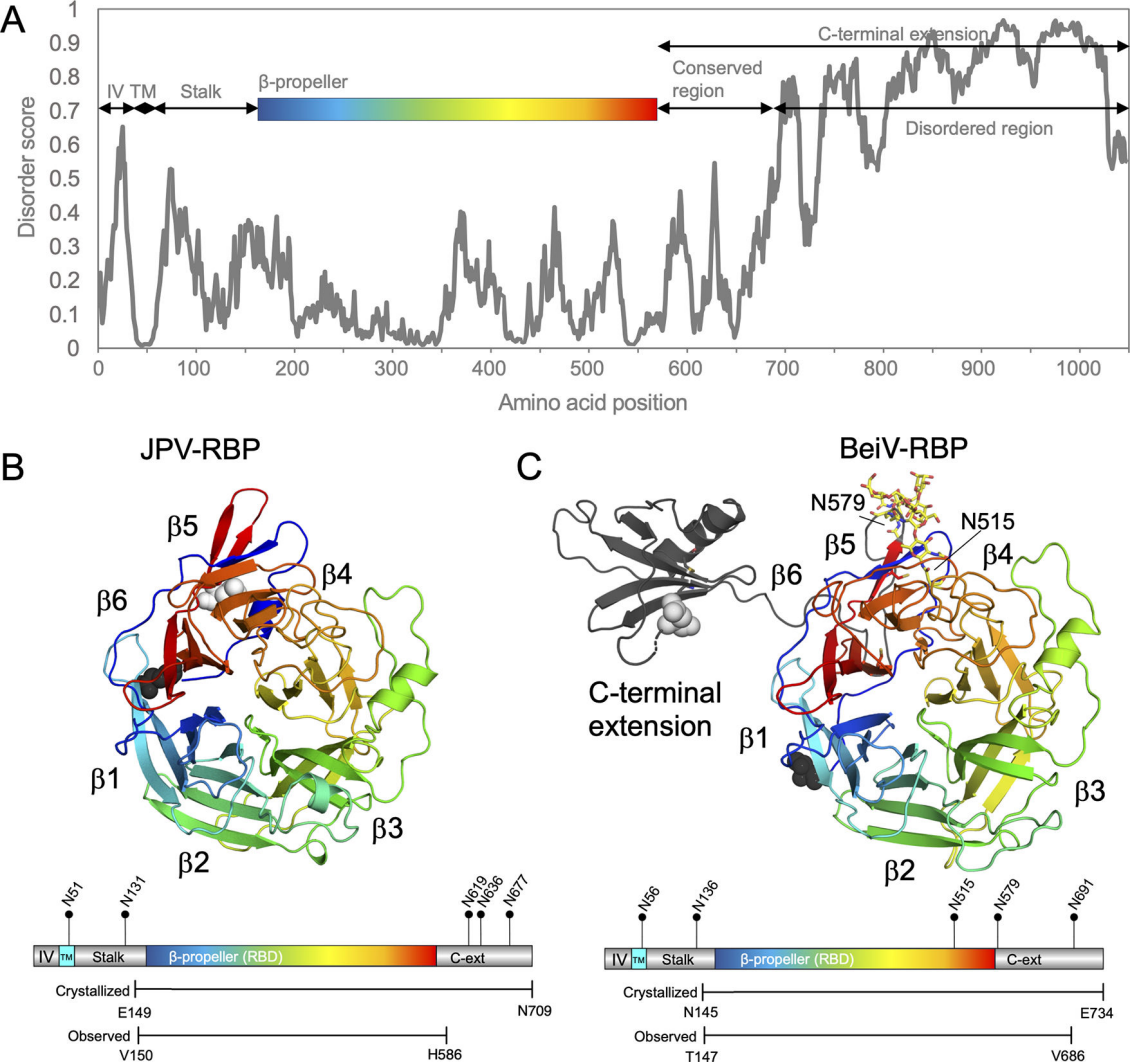
Structure of BeiV and JPV receptor-binding proteins. (**A**) The BeiV(rat)-RBP disorder score for each amino acid position, calculated using the “Prediction of Intrinsically Unstructured Proteins 2A” (IUPred2A) server (21), shows an increase in predicted disorder within the latter part of the C-terminal extension. The amino acid ranges corresponding to the intraviral regions (IV), transmembrane domain (TM), stalk region, six-bladed β-propeller, conserved and disordered regions of the C-terminal extension are indicated. (**B**) Crystal structure of the JPV-RBP β-propeller (JPV-RBP_β_). A protomer of the JPV-RBP_β_ dimer is shown in cartoon representation with blades labeled 1–6 (colored blue to red, from N- to C-terminus). Atoms comprising the N- and C-termini are shown as spheres and colored black and white, respectively. A gene diagram of JPV-RBP is shown below the structure, with the intraviral (IV, gray) region, transmembrane domain (TMD, light blue), stalk region (gray), and six-bladed β-propeller receptor-binding domain (rainbow) annotated. Predicted N-linked glycosylation sites (NXS/T, where X ≠ P) are marked with pins. The lengths of the crystallized and observed JPV-RBP construct are shown. (**C**) Crystal structure of the BeiV(HMC)-RBP β-propeller with C-terminal extension [BeiV(HMC)-RBP_β+_]. The structure and gene diagram are shown and colored as in panel A. N-linked glycosylation is colored yellow and presented as sticks.

The genomes of viruses within the genus *Jeilongvirus* are uncharacteristically long (up to 20 kb) when compared to other viruses in the order *Mononegavirales* (3, 8, 16). This is attributed, in part, to the presence of two unique open reading frames: a small hydrophobic (SH) protein and a transmembrane (TM) protein, which are thought to play roles in virulence (17) and cell-cell fusion (18), respectively. Additionally, *Jeilongvirus* RBPs feature a unique C-terminal extension of unknown structure and variable length (140–1,015 amino acids) (Fig. 1A; Fig. S1A). While little is known about the structure of this part of the RBP, the N-terminal region of the C-terminus encodes conserved cysteine residues, which may form local structure through disulfide bonding (Fig. S1B). Additionally, the C-terminal region of the C-terminus may exhibit O-linked type glycosylation and a high level of predicted overall structural disorder due to the high prevalence of proline, serine, and threonine (S/T/P) amino acid residues (19). Further reflective of the genomic variability of the jeilongvirus RBP, JPV-RBP, and a strain of BeiV-RBP isolated from human mesangial cells (HMC), lack the variable S/T/P-rich region and instead encode “RBP-associated” open reading frames enriched with S/T/P residues, termed “X” ORFs (19). Interestingly, variants of BeiV isolated from rats lack a stop codon at position 2,205 of the BeiV(rat)-RBP gene, resulting in an RBP of 1,046 amino acids, as opposed to the 734 observed from the laboratory-isolated BeiV(HMC) (20) (Fig. S1A).

Cellular and tissue tropism characteristics of a paramyxovirus are, in part, dictated by the interaction between an RBP and host-cell surface receptor (11, 22). Thus, a molecular-level understanding of paramyxoviral RBP architecture is essential when considering the determinants of interspecies transmission and zoonotic potential (22–24). Here, to address the paucity of knowledge about *Jeilongvirus* RBP function, we describe the crystal structures of the JPV-RBP β-propeller head domain (JPV-RBP_β_) and the BeiV(HMC)-RBP β-propeller head domain and previously undescribed C-terminal hat-like domain [BeiV(HMC)-RBP_β+_] to 2.2 and 3.5 Å resolution, respectively. This work broadens our appreciation of the pathobiological and architectural diversity of this understudied group of paramyxoviruses.

## RESULTS

### Structure determination and overview of JPV-RBP_β_ and BeiV(HMC)-RBP_β+_

Given the importance of paramyxoviral RBPs in dictating cellular tropism (11, 22), we sought to elucidate the structure of jeilongviral RBPs. To this end, constructs encoding the C-terminal six-bladed β-propeller head domain and extended C-terminus of JPV-RBP (Glu149-Asn709; termed “JPV-RBP_β+_”) and BeiV(HMC)-RBP [Asn145-Glu734; termed “BeiV(HMC)-RBP_β+_”] were recombinantly produced in the presence of the alpha-mannosidase inhibitor, kifunensine (25), and purified from human embryonic kidney (HEK) 293T cells (Fig. S2). Purified JPV-RBP_β+_ and BeiV(HMC)-RBP_β+_ were treated with endoglycosidase F1 (endoF1) (25), and crystallized using the vapor diffusion method (26). X-ray diffraction data were collected to 2.2 and 3.5 Å resolution, respectively. The JPV-RBP_β+_ structure was solved by molecular replacement using the structure of human parainfluenza virus 3 RBP (PDB: 1V21) as a search model. Subsequently, BeiV(HMC)-RBP_β+_ was solved using the refined structure of JPV-RBP_β+_ as a search model, where the majority of the BeiV(HMC)-RBP_β+_ C-terminal extension was visible (Lys576-Asn687).

Both JPV-RBP_β+_ and BeiV(HMC)-RBP_β+_ exhibit a six-bladed β-propeller fold characteristic of paramyxoviral RBP proteins, with each blade (β1 to β6) composed of four antiparallel β-strands (Fig. 1B and C, respectively). When superposed, the six-bladed β-propellers of JPV and BeiV RBPs align closely, with a calculated root-mean-square deviation (RMSD) of ~0.8 Å over 366 Cα atoms (Fig. S3A), with variation in the structures mostly contained in the loops and the flexible termini (Fig. S3B). In both BeiV(HMC)-RBP_β+_ and JPV-RBP_β+_ structures, electron density corresponding to the majority of the crystallized N-terminal stalk region (Pro146-Gln162 and Glu149-Met160, respectively) and β-propeller (Cys163-Cys575 and Cys161-Cys573, respectively) was well ordered. For BeiV(HMC)-RBP_β+_, a large portion of the C-terminal extension was visible (Lys576-Asn687). In contrast, many residues constituting the C-terminal extension of JPV-RBP_β+_ could not be built (Gly585-Asn709). The remaining C-terminal residues for both RBPs were disordered and directed toward the solvent channels in their respective crystals.

As the C-terminal extension of JPV-RBP_β+_ was not well ordered, the N-linked glycosylation was not observed at any of the three predicted N-linked glycosylation sequons within this region (Fig. 1B). Likewise, we do not observe a shift upon EndoF1 digestion (Fig. S2A and B), but it is not possible to know whether this is due to incomplete digestion or no occupancy at predicted sequons. Within the ordered region of the β-propeller of BeiV(HMC)-RBP_β+_, there are two predicted N-linked glycosylation sequons, Asn515 and Asn579 (Fig. 1C). We observe a partially ordered N-linked glycan at residue Asn515 BeiV(HMC)-RBP_β+_ on both chains (Fig. S7A), which is consistent with our SEC and SDS-PAGE analysis following EndoF1 treatment (Fig. S2C and D), where we did not observe a shift in protein mass, indicating that the chitobiose core of the glycan was likely inaccessible. This N-linked glycosylation is positioned at the pinnacle of loop β5L34 and is packed against the β-sheet formed by residues of the N-terminus and C-terminal extension, which potentially have a role in stabilizing and protecting the glycan from the enzyme (Fig. S7A). Furthermore, electron density at residue Asn579 in one chain of the asymmetric unit enabled the partial building of N-linked glycosylation at the beginning of the C-terminal extension (Fig. S7A).

**Fig 2:**
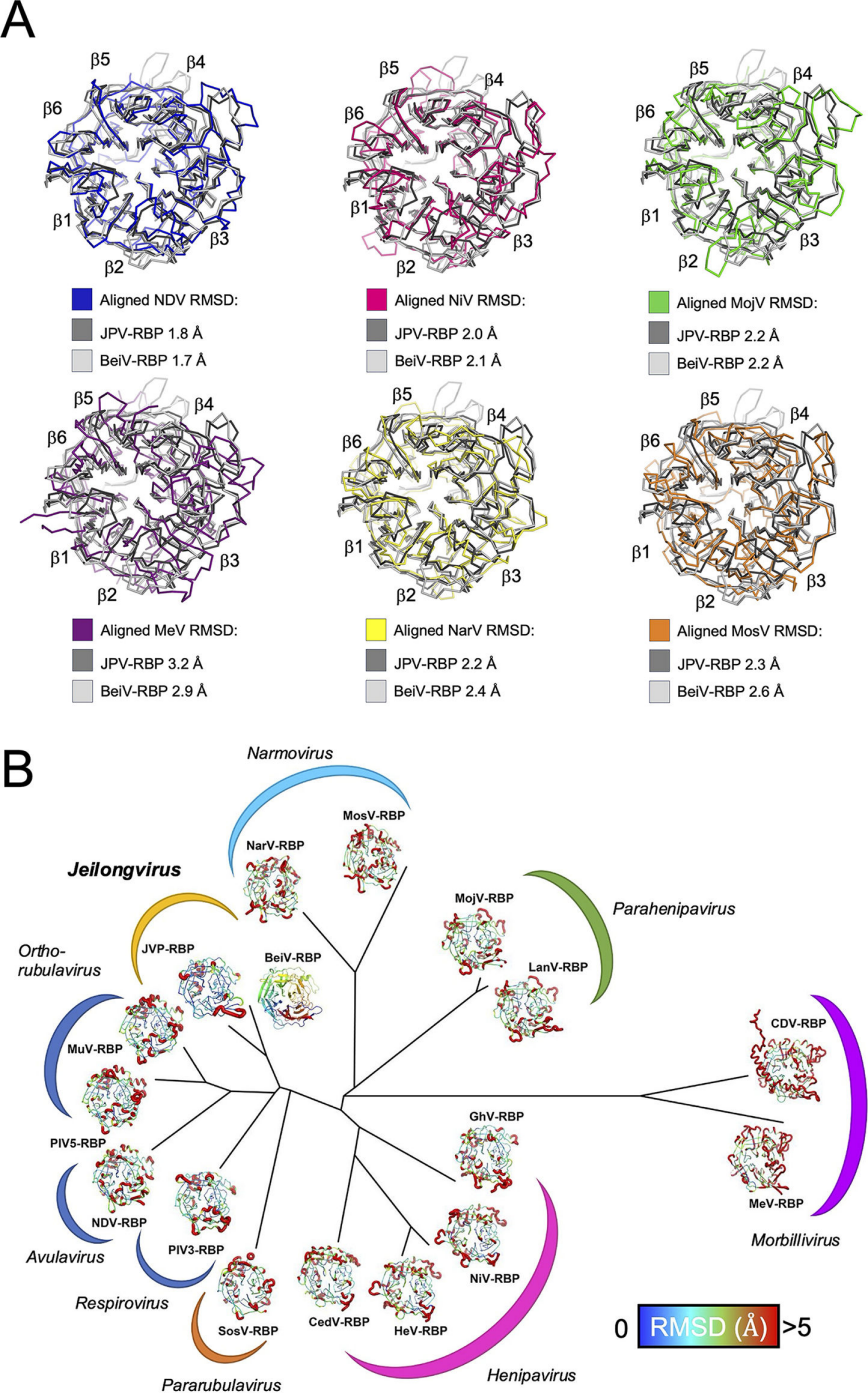
The BeiV-RBP and JPV-RBP β-propellers form a singular structural class. (**A**) Structure overlays of JPV-RBP_β_ (dark gray) and BeiV(HMC)-RBP_β_ (light gray) with other paramyxoviral RBP structures: from left to right, top to bottom, NDV, Newcastle disease virus (blue, 1E8V) (27); NiV, Nipah virus (pink, 2VWD) (28); MojV, Mojiang virus (green, 5NOP) (29); MeV, measles virus (purple, 2ZB5) (30); NarV, Nariva virus (yellow, 7ZM6) (31); and MosV, Mossman virus (orange, 7ZM5) (31). RMSDs are annotated. (**B**) Structure-based phylogenetic analysis of paramyxoviral RBP monomers: JPV; BeiV; PIV3, parainfluenza virus 3 (1V2I) (32); SosV, Sosuga virus (6SG8) (33); NDV (1E8V) (27); PIV5, parainfluenza virus 5 (4JF7) (34); MuV (5B2C) (35); MojV (5NOP) (29); GhV, Ghana virus (4UF7) (36); NiV (2VWD) (28); HeV, Hendra virus (2X9M) (37); CedV, Cedar virus (6THB) (38); MeV (2ZB5) (30); LANV, Lanya virus (8K80) (39); CDV, Canine distemper virus (7ZNY) (40); NarV, Nariva virus (7ZM6) (31); and MosV, Mossman virus (7ZM5) (31). The Structural Homology Program (41) was utilized to calculate evolutionary distance matrices by pairwise superposition of RBP structures. The resultant matrices were used to plot an unrooted tree in PHYLIP (42). BeiV(HMC)-RBP_β+_ is displayed in cartoon representation and colored as a rainbow from N-terminus (blue) to C-terminus (red). The remaining RBPs are displayed as cartoon putty, where RMSD with respect to the structure of BeiV(HMC)-RBP is represented by a color scale and the thickness of the chain, with blue/thin illustrating the least RMSD and red/thick the largest RMSD between equivalent Cα atoms of the two structures. Arcs are displayed to highlight the genus-specific groupings of the RBPs. The arcs of avula, respiro, and orthorubulaviruses are colored dark blue to reflect their sialic acid receptor usage.

Of the previously characterized paramyxoviral RBP β-propellers, JPV-RBP_β+_ and BeiV(HMC)-RBP_β+_ display a pattern of disulfide bonding comparable to and most closely structurally aligned with HN-type RBPs, including Newcastle disease virus (NDV)-RBP_β_ (Fig. 2A and B). In contrast, JPV-RBP_β+_ and BeiV(HMC)-RBP_β+_ exhibit a more distant structural relationship with henipaviral, henipa-like, morbilliviral, and the narmoviral RBPs (Fig. 2A and B). Furthermore, both JPV-RBP_β+_ and BeiV(HMC)-RBP_β+_ β-propellers show no conservation with morbillivirus and the henipavirus RBPs at known receptor-binding sites (Fig. S4), suggesting that they are unlikely to exhibit shared receptor usage.

### BeiV-RBP and JPV-RBP encode residues required for sialic acid entry and hydrolysis

**Fig 3:**
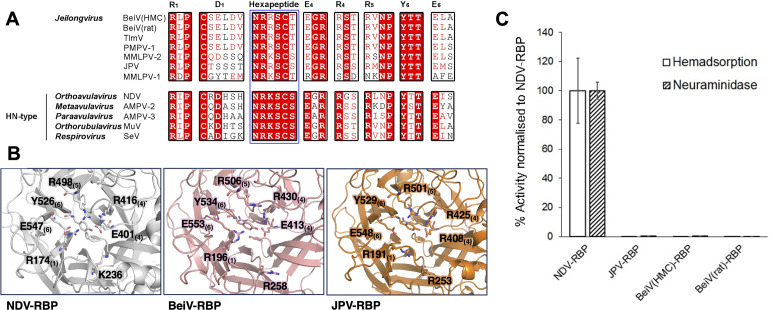
Regions known to confer sialic acid binding and hydrolysis functionality. (**A**) Alignment of the RBP amino acid sequences from BeiV(HMC), BeiV(rat), Tailam virus (TlmV), Pohorje Myodes paramyxovirus 1 (PMPV-1), Mount Mabu Lophuromys paramyxovirus 1 and 2 (MMLPV-1/2), J paramyxovirus (JPV), Newcastle disease virus (NDV), avian paramyxoviruses 2 and 3 (AMPV-2/3), mumps virus (MuV), and Sendai virus (SeV). Residues associated with primary sialic acid interactions, including the seven conserved sialidase residues (14) and hexapeptide motif (15), are labeled according to residue and blade location and annotated above alignments. (**B**) A close-up of the NDV-RBP β-propeller (27) (gray, left) with the side chain residues constituting the sialic acid binding site annotated and shown as sticks, with oxygen atoms colored red, carbon atoms gray, and nitrogen atoms blue. The equivalent position of BeiV(HMC)-RBP_β+_ (pink—center) and JPV-RBP_β_ (orange—right) is shown with the hypothetical position of the primary sialic acid binding site. β-propellers are rendered in cartoon representation, and important residues are annotated and shown using stick representation. (**C**) JPV-RBP, BeiV(HMC)-RBP, and BeiV(rat)-RBP neuraminidase (49) and hemadsorption (50) (activity normalized to cell surface expression and an NDV-RBP control). For both hemadsorption (*n* = 12) and neuraminidase (*n* = 6) assays, error bars represent the standard deviation across all replicates.

Although previous studies indicate that JPV-RBP lacks hemagglutinin and hemadsorption activity (1, 3), Jeilongviral RBPs display many of the conserved residues required to interact with sialic acid that are found in other HN-type paramyxoviral RBPs (27, 32, 35, 43), including six of the seven sialidase residues: Arg_1_, Glu_4_, Arg_4_, Arg_5_, Tyr_6_, and Glu_6_ (Fig. 3A and B) (subscript refers to location on blades 1–6 of the β-propeller fold) (14, 27, 44). Both JPV-RBP and BeiV-RBP (HMC and rat origin) lack Asp_1_, where this residue is replaced with Ser or Glu, respectively. Interestingly, however, Asp_1_ may not be essential, as observed in the interaction between 3′-sialylactose (3′-SL) and the RBP of mumps virus (MuV) RBP (35). Additionally, JPV-RBP and BeiV-RBP (HMC and rat origin) exhibit a variant hexapeptide motif (Asn-Arg-Lys-Ser-Cys-Ser), where an Arg is presented at the third position as opposed to a Lys. In both our JPV-RBP_β+_ and BeiV(HMC)-RBP_β+_ crystal structures, residues from both the sialidase and hexapeptide motifs adopt conformations that largely match those of NDV-RBP (Fig. 3A). Additionally, JPV-RBP_β+_ and BeiV-RBP_β+_ retain either identical or physicochemically similar residues associated with a secondary sialic acid binding site implicated in triggering fusion for NDV-RBPs (Fig. S5A) (45–48). Further, this site is unobstructed by the C-terminal extension. However, despite the presence of these residues and consistent with previous findings (1, 3), HEK 293T cells displaying either full-length (IV-TM-Stalk-head-hat) JPV-RBP, BeiV(HMC)-RBP, and BeiV(rat)-RBP lacked hemadsorptive and neuraminidase activities under the conditions tested, when compared to an NDV-RBP control (Fig. 3C) (49, 50). Interestingly, this second sialic acid site has not been observed in all HN-type RBP structures (12, 13, 43, 51), indicating that paramyxoviral sialic acid functionality is not dependent upon its existence.

### Extension of the homodimeric interface provides a structural basis for the absence of observed hemadsorption and neuraminidase activity

**Fig 4:**
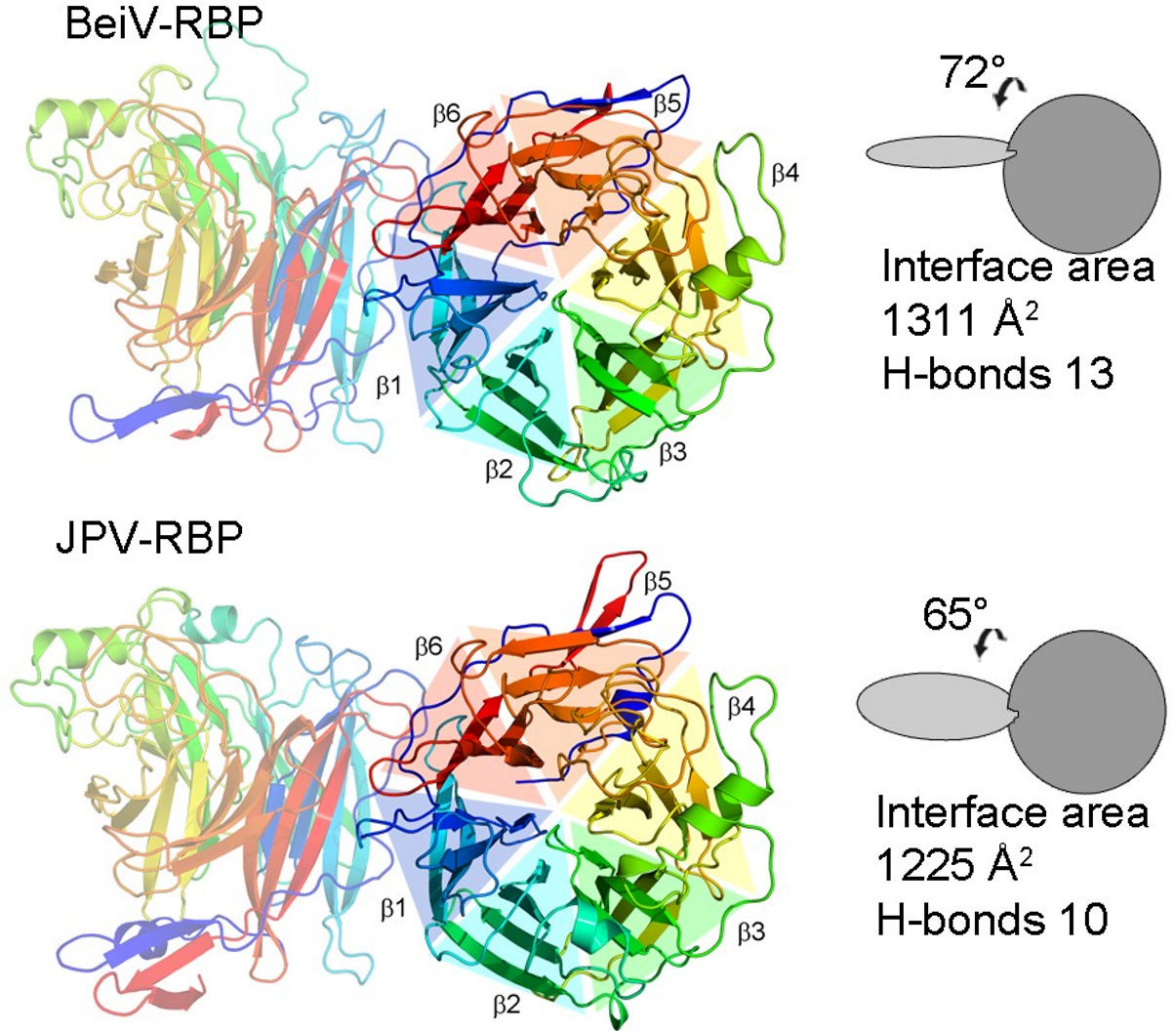
Comparison of JPV-RBP_β_ and BeiV(HMC)-RBP_β+_ dimers. Crystallographically observed JPV-RBP_β_ and BeiV(HMC)-RBP_β+_ dimers are shown in cartoon representation with monomers colored rainbow from the N-terminus to C-terminus (blue to red, respectively). The calculated angle of association, interface area, and number of hydrogen bonds are displayed on the right-hand side of the relevant dimer.

Two near-identical β-propeller head domains form dimers in the asymmetric units of JPV-RBP_β+_ (RMSD of ~0.2 Å over 435 Cα atoms) and BeiV(HMC)-RBP_β+_ (RMSD of ~0.7 Å over 527 Cα atoms). Similar to other paramyxovirus RBPs (31, 33, 37), the interfaces between JPV-RBP_β+_ and BeiV(HMC)-RBP_β+_ monomers are mediated through the interaction between blades β1 and β6 and occlude ~1,230 and ~1,310 Å^2^ of solvent accessible surface area (52), respectively (Fig. 4). The angle of association between JPV-RBP_β+_ and BeiV(HMC)-RBP_β+_ monomers is ~65° and ~72°, respectively (Fig. 4), which is similar to the HN-type RBPs, with angles ranging from ~53° to 64° (31, 33).

**Fig 5:**
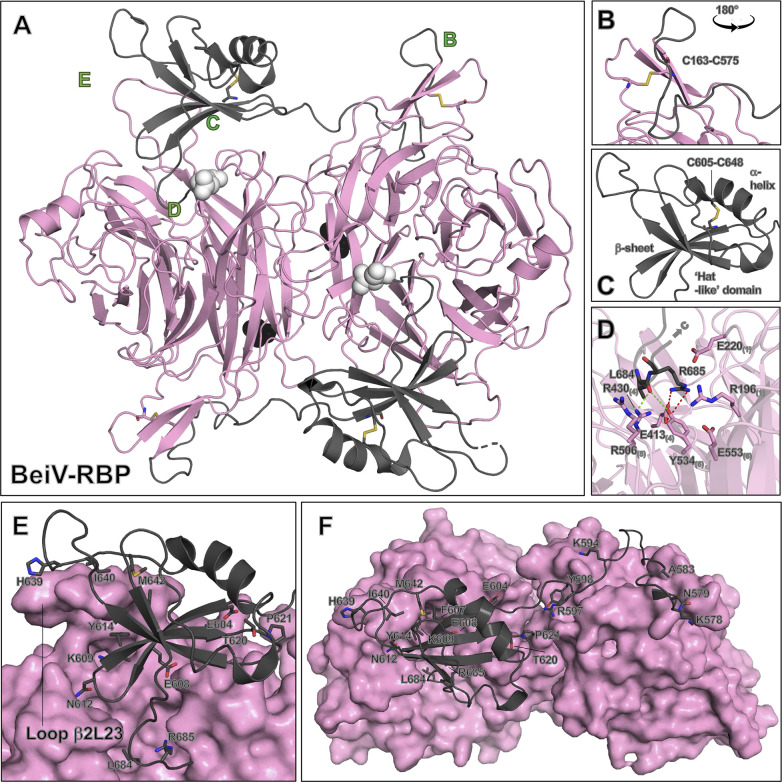
Organization of the BeiV-RBP C-terminal extension. (**A**) The BeiV(HMC)-RBP_β+_ dimer is shown in cartoon representation with the β-propeller head-domain colored pink and the “hat-like” domain colored gray. The letters B, C, D, and E show the location of the features highlighted in the zoom-in panels. Key disulfide bonds highlighted in the zoom-in panels are also shown as sticks in panel A. The termini of the resolved structure are shown as spheres, with the N-terminus colored black and the C-terminus colored white. (**B**) Panel B is a zoom-in of the beginning of the C-terminal extension. This is rotated 180° horizontally with respect to panel A. The C-terminal extension forms a β-ribbon with residues from the N-terminus of the BeiV(HMC)-RBP_β+_ structure. This is stabilized by a disulfide (Cys163-Cys575), with mainchain and sidechains shown as sticks. (**C**) The hat-like domain in isolation is rendered as a cartoon. The β-sheet, α-helix, and disulfide bond (Cys605-Cys648), which stabilizes the structure of the hat-like domain, are annotated. The main chain and side chains of residues C605 and C648 are shown as sticks. (**D**) Interaction of the C-terminal “hook-like” extension (gray) with the putative sialic acid binding site (pink), rendered in cartoon representation. The conserved sialic acid binding residues interact with the main chain of the hook and the side chains of residues Leu684 and Arg685. The main chain and side chains of key residues are shown as sticks. Hydrogen bonds are shown as green dashed lines and salt bridges as red dashed lines. (**E**) The top face of BeiV(HMC)-RBP_β+_, where loop β2L23 interacts with the hat-like domain, is rendered as a surface (pink). The “hat-like” domain is shown in cartoon representation (gray) with residues involved in H-bonding and salt-bridge formation shown as sticks (residues identified using PDBePISA analysis). (**F**) The dimeric head domain of BeiV(HMC)-RBP_β+_ is rendered as a surface (pink). The linker of the C-terminal extension (gray), represented using a cartoon, traverses across the dimeric interface. Analysis using the PDBePISA server (52) reveals many residues of the C-terminal extension are involved in H-bonding and salt-bridge formation, including residues within this linker region. The main chain and side chains of relevant residues are shown as sticks.

While the majority of the JPV-RBP_β+_ extended C-terminus (Gly585-Asn709) is disordered, the elongated C-terminus of BeiV(HMC)-RBP_β+_ is largely observable in the crystal (Lys576-Asn687) (Fig. 1C) and increases the extent of the homodimeric interaction to ~4,250 Å^2^. Similar to the RBP from NDV Ulster strain (NDV-RBP^Ulster^) (Fig. S5B), C-terminal residues of BeiV(HMC)-RBP_β+_ occlude residues implicated in sialic acid recognition and hydrolysis in other HN RBPs (Fig. 5D and E), providing a structural basis for the absence of observed hemadsorptive and neuraminidase activity (Fig. 3C). The BeiV(HMC)-RBP_β+_ C-terminal extension exchanges a hat-like domain with the adjoining protomer of the homodimer (Fig. 5A), a process termed “domain-swapping,” where identical monomers switch domains with one another (53). Although reminiscent, the occlusion of the potential receptor-binding site in the BeiV(HMC)-RBP_β+_ homodimer differs from that observed in the avirulent NDV-RBP^Ulster^. Indeed, the hat-like domain of BeiV(HMC)-RBP_β+_ forms a structure that resembles a blade from the six-bladed β-propeller fold. However, instead of a hat-like domain, NDV-RBP^Ulster^ bears a shorter, 45 amino acid C-terminal peptide (Fig. S1A and B), which extends along the outside of the β-propeller before forming an α-helix that is stabilized at the top of the dimeric interface through an interchain disulfide bond (Fig. S5B) (48). Following the α-helix, the chain terminates in the sialic acid binding site of the same protomer, providing a mechanism of autoinhibition (48). In contrast, the C-terminal extension of BeiV(HMC)-RBP_β+_ does not encode an interchain disulfide, and it forms the much more substantial hat-like domain that recognizes the putative sialic acid binding site of the adjacent protomer.

### The jeilongvirus hat-like domain

The BeiV(HMC)-RBP_β+_ hat-like domain is unique amongst all reported paramyxoviral RBP structures and consists of an α-helix, a β-sheet composed of four antiparallel β-strands, and a long anchoring arm, which buries into the putative sialic acid binding cavity (Fig. 5A). Residues Leu573-Gly577 of the C-terminus form a β-sheet with residues Met168-Leu172 of the N-terminus and are conformationally reinforced by a disulfide bond (Cys163-Cys575) (Fig. 5B). A long linker composed of residues Lys578-Glu604, which is observed to be glycosylated at position Asn579 on one chain in asymmetric unit (Fig. S7A), traverses the dimer interface. A well-conserved intra-domain disulfide bond Cys605-Cys648 secures the position of the hat-like domain (Fig. S1B; Fig. 5C). Several salt bridges and an extensive hydrogen bonding network form between the linker and residues of the C-terminus, N-terminus, and the pinnacles of loops β6L12 and β1L34, further comprising the interaction between the hat-like domain and the β-propeller (Fig. S6).

The hat-like domain of BeiV(HMC)-RBP_β+_ displaces loop β2L23 of the propeller (Fig. S3B and S5), enabling projection of the C-terminal hook of the hat-like domain into the top center of the β-propeller (Fig. 5D and E). This interaction is stabilized by hydrogen bonds and salt bridges formed with residues of loops β2L01, β3L01, and β3L23 (Fig. S6), as well as interactions between the main chain carbonyl groups and the positively charged surfaces of the RBP. For example, Arg685 of the hat-like domain forms a salt bridge with Glu413_(4)_, a residue associated with sialidase activity (Fig. 5D). Additionally, Arg430_(4)_ of the triarginyl motif forms a hydrogen bond with the main chain carbonyl of Leu684 of the “hook” shaped C-terminus.

Although not well ordered enough to model, electron density putatively corresponding to residues C-terminal to Asn687 of BeiV(HMC)-RBP_β+_ of the hat-like domain is observed. Indeed, electron density observed between the BeiV(HMC)-RBP_β+_ hat-like domain of one protomer and the BeiV(HMC)-RBP_β+_ hat-like domain of its symmetry mate (Fig. S7B) provides evidence for further, albeit possibly weaker and more transient interactions between the C-terminus and the rest of the BeiV(HMC)-RBP_β+_. In the case of JPV-RBP_β+_, although the C-terminal extension was not ordered in the structure, a short portion of electron density potentially corresponding to the Cα backbone (Fig. S7C [53]) is bound within the putative sialic acid site. This density is proximal to Arg191_(1)_, Glu408_(4)_, Arg425_(4)_, Arg501_(5)_, Tyr529_(6)_, Glu548_(6)_, and Arg557, and Arg253 of the variant hexapeptide motif, and coincides with the electron density visible within the BeiV(HMC)-RBP_β+_ putative active site (Fig. S7D). Consistent with the low level of order and occupancy of residues in this region of the C-terminal extension, loop β2L23 of JPV-RBP_β+_ is not displaced as it is in the BeiV(HMC)-RBP_β+_ structure (Fig. S3B), and instead forms a well-ordered α-helix proximal to the putative sialic acid binding site, as observed in other paramyxoviral HN-type RBPs (27, 29, 30, 32, 35, 36, 38, 43, 54). Furthermore, the superposition of the hat-like domain from BeiV(HMC)-RBP_β+_ onto JPV-RBP_β+_ reveals that loop β2L23 would sterically obstruct the positioning of the ordered β-sheet of the hat-like domain.

While we do not observe ordered electron density for the majority of the C-terminal extension of JPV-RBP_β+_ in the crystal structure, an AlphaFold3 (55) prediction of the C-terminal extension of JPV-RBP, generated from a prediction of two copies of the full-length sequence, bears a striking resemblance to the BeiV(HMC)-RBP_β+_ hat-like domain (Fig. S8A). Indeed, despite the low sequence identity across the C-terminal extension (Fig. S1A and B), the AlphaFold3 prediction of the JPV-RBP hat-like domain aligns moderately well, with an RMSD of 2.2 Å (over 70 aligned Cα residues). Furthermore, the model exhibits a similar placement of the hat-like domain at the top central region of the six-bladed β-propeller to that observed in BeiV(HMC)-RBP_β+_, suggestive of similar modes of interaction. A search with Foldseek (56) reveals that similarly, the BeiV(HMC)-RBP_β+_ hat-like domain exhibits the greatest expected structural similarity to C-terminal regions of other jeilongvirus receptor-binding proteins, as predicted by Colabfold (57) in the Big Fantastic Virus Database (58). Additionally, this search revealed a low-confidence similarity with an uncharacterized monkey poxvirus protein, B15L. Furthermore, the hat-like domain of BeiV(HMC)-RBP_β+_ had numerous low-confidence matches with the ATP-binding N-terminal lobe of protein kinase domain-containing (PKDC) proteins from diverse species (59). For example, the BeiV(HMC)-RBP_β+_ hat-like domain has the same topology as the N-terminal lobe of a PKDC protein from Glycine max (Fig. S8B and C). However, α-helix 1 of the hat-like domain differs in its position relative to the β-sheet, where the BeiV(HMC)-RBP_β+_ hat-like domain α-helix rests on the concave side of the β-sheet. While interesting, the functional relevance of similarity to PKDCs is likely limited as the hat-like domain of BeiV(HMC)-RBP_β+_ lacks the GxGxxG motif (ATP-binding loop) and the b3 Lys present in PKDC proteins (59). Thus, although it is possible that BeiV(HMC)-RBP_β+_ may exhibit some ATP-binding functionality, as has been shown previously for pseudokinases that have degraded canonical kinase ATP-binding motifs, the RBP is unlikely to exhibit kinase or ATP-catalysis functionality. While the evolutionary basis for how the hat-like domain became a part of the paramyxovirus RBP repertoire remains a mystery, these findings provide new insights into the possible relationship of the RBP with ancestral proteins.

## DISCUSSION

Jeilongviruses constitute a group of emerging and poorly understood paramyxoviruses. Here, we provide molecular-level insights into the unique architecture of the jeilongvirus RBP, a cellular and species-tropism-dictating protein. Our molecular-level analysis of JPV-RBP_β+_ and BeiV(HMC)-RBP_β+_ clarifies the structural relationship of these jeilongviral proteins with other paramyxoviral RBPs. Indeed, consistent with genetic analysis and the near-conservation of residues required for sialic acid binding and hydrolysis, both at the primary (14, 15) (Fig. 3A and B) and putative secondary (45–47) binding sites (Fig. S5A), we find that both JPV-RBP_β+_ and BeiV(HMC)-RBP_β+_ bear the closest structural relationship with HN-type RBPs, including respiroviruses, orthoavulaviruses, and rubulaviruses (Fig. 2A and B).

Intriguingly, we were unable to observe sialic acid binding and hydrolysis activity for these glycoproteins under the conditions tested (Fig. 3C). Our structures provide a molecular-level rationale for the absence of this observed activity. Indeed, we find that the elongated C-terminus of the jeilongvirus RBP (Fig. S1A and B), which may have possibly arisen from gene duplication of a β-propeller blade or acquisition of a host protein (Fig. S8B and C), extends toward the membrane distal region of the adjacent protomer of the β-propeller homodimer and forms a unique hat-like domain that sterically impedes the putative sialic acid-binding site (Fig. 5). The presence of the unique C-terminus, combined with the high level of sialidase and hexapeptide motif conservation (Fig. 3A and B), suggests that jeilongviral RBPs are likely able to regulate sialic acid binding and hydrolysis activity. Furthermore, the observation that the hat-like domain is differentially ordered in the crystal structures of JPV-RBP_β+_ and BeiV(HMC)-RBP_β+_ supports that this region of the protein may regulate activity by only being transiently associated with the sialic acid-binding site (Fig. 1; Fig. S7C and D). While the cell type, host-cell factors, or virus replication stage that may modulate this putative autoinhibitory activity remain unknown, such a hypothesis is consistent with our observation that the polypeptide corresponding to the otherwise disordered C-terminus of JPV-RBP_β+_ appears to occupy the putative sialic acid-binding site (Fig. S7C). As it is likely that our hemadsorption and neuraminidase assays do not replicate the environment relevant to native rodent host-cell infection, further studies are required to fully dissect the restriction factors and conditions necessary to reconstruct jeilongvirus RBP activity. To assess whether removal of the C-terminal region hat-like domain may regulate jeilongvirus RBP sialic acid-associated activity (e.g., via proteolytic cleavage by an unknown protease), we attempted to produce constructs of BeiV-RBP_β_ and JPV-RBP_β_ that lacked the C-terminal hat-like domain. However, unlike our construct containing the C-terminal extension, our truncated constructs were heterogeneous in solution (JPV-RBP_β_) or did not express to a level necessary for protein purification (BeiV-RBP_β_), indicative that further construct screening is required to produce truncated jeilongvirus RBPs suitable for functional studies. Future interrogation of this hypothesis would benefit from assessment of jeilongvirus RBP maturation in a range of rodent cells and determination of the physicochemical conditions necessary to (e.g., pH and temperature) modulate RBP functionality, *in vitro*. We also demonstrate that the region of the jeilongvirus RBP that aligns both structurally (Fig. S5A) and by sequence to the position of the secondary binding site in the RBP of NDV is accessible and not occluded by the C-terminal extension. Given a lack of observed hemadsorption activity, it appears that this region, which has also been implicated in fusion activation in NDV (46), also does not interact with sialic acid interactions under the conditions tested.

How obstruction of the putative sialic acid-binding site by the C-terminal hat-like domain could regulate the jeilongvirus RBP activity remains a mystery. In the case of the avirulent Newcastle disease virus Ulster strain RBP (NDV-RBP^Ulster^), the C-terminal extension modulates pathogenicity (48, 60) (Fig. S5B). The extended C-terminus of NDV-RBP^Ulster^ both occludes the receptor-binding site of its own protomer in the dimer and exhibits an inter-subunit disulfide bond that regulates HN activity and dimerization. While the C-terminal extensions of BeiV(HMC)-RBP and NDV-RBP^Ulster^ bear no apparent relationship in sequence, length, or structure (Fig. 5A; Fig. S1A, B, and S5B), it is possible that, similar to NDV-RBP^Ulster^, jeilongvirus RBPs may also require proteolysis at the C-terminus to recognize and hydrolyze sialic acid (61). Although we did not identify a conserved proteolysis motif, such as that found in NDV-RBP^Ulster^ (i.e., ^572^Lys-Glu-Ala-Lys^575^) (61), analysis with the EXPASY PeptideCutter tool (62) indicates that the JPV-RBP or BeiV(HMC)-RBP C-terminal extension may encode multiple potential cleavage sites for proteases, including trypsin, chymotrypsin, elastase, and thermolysin (63). Future investigations into whether the hat-like domain is shed via such a mechanism to establish sialic acid binding and hydrolysis activity would allow assessment of whether jeilongviruses utilize a universal approach toward autoinhibition, as well as whether such protease sensitivity is a restriction factor specific to the host rodent reservoir species. Moreover, the distinctiveness of the structure and length of the extended jeilongviral C-termini, combined with the observation that only a subset of NDV strains bear an elongated C-terminus on their RBP that modulates activity (Fig. 5A; Fig. S1A, B, and S5B) (64), suggests that the extended C-termini of jeilongviruses and avirulent NDV strains arose independently over the course of their evolution from common sialic acid-binding ancestral paramyxoviruses.

Emerging and re-emerging viruses continue to make a profound impact on human health and the economy (65, 66). Although the economic, environmental, and biomedical importance of jeilongviruses remains unclear, this does not diminish the potential impact of these or related paramyxoviruses in the future. Furthermore, although much work is needed to understand the tropism and replication characteristics of jeilongviruses, the detailed understanding of RBP structure and function delivered by this study constitutes an essential building block that will help us decipher pathobiological determinants of interspecies transmission at a molecular level. Indeed, by increasing our understanding of the structural repertoires available to paramyxovirus RBPs, this work strengthens our preparedness for the potential emergence of these and other paramyxoviruses from native reservoirs into new species, including humans.

## MATERIALS AND METHODS

### Protein production

JPV-RBP_β+_ and BeiV(HMC)-RBP_β+_ constructs were generated from human codon-optimized genes of the full-length RBPs (Genbank NC_007454.1 and YP_512253.1, respectively). JPV-RBP_β+_ (Glu149-Asn709) and BeiV(HMC)-RBP_β+_ (Asn145-Pro1046) were cloned into the pHLsec mammalian expression vector, which encodes a C-terminal hexahistidine tag (67). Proteins were produced by transient transfection of HEK 293T cells in the presence of 5 µM kifunensine (25), and secreted protein was harvested after 72 h incubation at 37°C, 5% CO_2_. Cell supernatant was exchanged into 10 mM Tris (pH 8.0), 150 mM NaCl, and concentrated using an ÄKTA Flux diafiltration system (Cytiva). Immobilized metal-affinity chromatography was then performed with a HisTrap HP (Cytiva) column and eluted using 250 mM imidazole. Endoglycosidase F1 (EndoF1) was used to cleave (10 µg/mg protein, 12 h, 21°C) any existing N-linked glycans at the di-N-acetylchitobiose core. Size-exclusion chromatography was performed in 10 mM Tris, pH 8.0, 150 mM NaCl buffer using a Superdex 200 10/30 column (Cytiva).

### Crystallization and X-ray diffraction data collection

JPV-RBP_β+_ crystals were grown using the nanoliter-scale sitting-drop vapor-diffusion method at room temperature, using 100 nL protein (3.5 mg/mL) and 100 nL precipitant (26). Crystals grew in 0.1 M carboxylic acid, 0.1 M tris/bicine pH 8.5, 6% sucrose, 0.2 M ammonium sulfate, 37.5% Morpheus (Molecular dimensions) precipitant mix 4, consisting of 25% vol/vol 2-methyl-2,4-pentanediol (MPD), 25% wt/vol PEG 1000 (P1k) and 25% wt/vol polyethylene glycol 3350 (PEG 3350). The crystal was immersed in a solution consisting of the precipitant with an additional 10% MPD, prior to cryo-cooling. X-ray diffraction data were collected at a wavelength of 0.9282 Å on beamline I04-1, Diamond Light Source (DLS), Didcot, UK. Reflections were indexed, integrated, and scaled using the xia2 package (68) (Table S1), and molecular replacement was performed using PHASER (69), the human parainfluenza virus 3 RBP (PDB: 1V21) (32) as a search model. COOT (70) was used for model building, Phenix (71) for refinement. Structure validation was performed using Molprobity (72).

BeiV(HMC)-RBP_β+_ crystals were grown using nanoliter-scale sitting-drop vapor-diffusion at room temperature, using 100 nL protein (3.2 mg/mL) and 100 nL precipitant (26). Crystals grew in 0.2 M potassium thiocyanate, 0.1 M sodium cacodylate, pH 6.5, 8% wt/vol poly-γ-glutamic acid polymer, 6% 1,5-diaminopentane di-HCl. Crystal cryo-cooling was performed in a solution consisting of reservoir solution with an additional 20% glycerol, prior to X-ray data collection, which was performed at a wavelength of 0.9282 Å on beamline I04-1, DLS. Reflections were indexed, integrated, and scaled using the xia2 package (68) (Table S2) and molecular replacement using PHASER (69) with the JPV-RBP_β_ structure as a search model. COOT (70) was used for model building, Phenix (71) for refinement. Structure validation was performed using Molprobity (72). Density modification using Parrot (73) was performed to aid in the building of this structure.

### Hemadsorption and neuraminidase assays

Full-length (IV-TM-Stalk-head-hat) JPV-RBP (Met1-Asn709, Genbank: YP_338084.1) and BeiV(HMC)-RBP (Met1-Pro1046, Genbank: YP_512253.1), and BeiV(rat)-RBP (Met1-Glu734, Genbank: AOV81769.1) were cloned into the pHLsec vector with a C-terminal hexa-histidine tag (67) and transfected with Lipofectamine 2000 (ThermoFisher, product no. 11668030) into HEK 293T cells. The hemadsorption method to determine sialic acid binding was adapted from Morrison and McGinnes (50). HEK 293T cell monolayers were washed with phosphate-buffered saline (PBS), pH 7.4 (with MgCl_2_ and CaCl_2_), 24 h following transfection, prior to incubation with 2% sheep blood (Thermo Scientific Oxoid, 12967755) at 4°C for 30 min. Cells were gently washed to remove unadsorbed erythrocytes, prior to lysing absorbed erythrocytes using 50 mM Tris, pH 7.4, 5 mM ethylenediaminetetraacetic acid, 150 mM NaCl, and 0.5% Nonidet P-40. Absorbance at 540 nm was measured using a CLARIOStar plate reader (BMG Labtech). Assays were normalized to the NDV-RBP positive control and the relative level of protein cell surface expression, as assessed by enzyme-linked immunosorbent assay.

Neuraminidase activity was determined through hydrolysis of the substrate 2′-(4-methylumbelliferyl)-α-d-N-acetylneuraminic acid (MU-Neu5Ac; Sigma-Aldrich, product no. M8639), as described previously (49, 74). Twenty-four hours following transfection, HEK 293T monolayers were washed with PBS, pH 7.4, counted, and seeded in a 96-well black nontransparent plate at a density of 25,000 cells per mL. Cells were pelleted by spinning at 1,500 rpm for 5 min, and the supernatant was replaced with 0.1 M sodium acetate, pH 6.0 containing 1 mM MU-Neu5Ac. The plate was incubated for 1 h at 37°C prior to the addition of 0.25 M glycine buffer, pH 10.7 to stop the reaction. The amount of free 4-methylumbelliferone was fluorometrically determined at 365 nm for excitation and 450 nm for emission using a CLARIOStar plate reader (BMG Labtech).

### Structure-based phylogenetic analysis

To perform structural phylogenetic analysis of the paramyxoviral RBP β-propellers, water molecules, ligands, and protein residues outside of the canonical fold were removed from the six-bladed β-propeller monomers. Pairwise distances between RBP structures were calculated using the Structural Homology Program (41, 75, 76). In PHYLIP, pairwise evolutionary distance matrices were used to generate an unrooted phylogenetic tree (42).

## Data Availability

The atomic coordinates and structure factors for JPV-RBP_β+_ and BeiV(HMC)-RBP_β+_ have been deposited in the Protein Data Bank with the accession codes 9QD1 and 9QD0, respectively.

## SUPPLEMENTAL MATERIAL

Supplemental material mbio.01501-25-s0001.docx Supplemental tables and figures.

ASM does not own the copyrights to Supplemental Material that may be linked to, or accessed through, an article. The authors have granted ASM a non-exclusive, world-wide license to publish the Supplemental Material files. Please contact the corresponding author directly for reuse.
