# Supplementary material for: Molecular basis for occlusion of the jeilongvirus receptor-binding site by the elongated C-terminus
Source: mBio. 2025 Nov 25;17(1):e01501-25. doi: 10.1128/mbio.01501-25 (PMC12802151; doi:10.1128/mbio.01501-25)
Supplement: Supplemental material — Supplemental tables and figures. [file mbio.01501-25-s0001.docx]

Supplementary Figures and Methods for:

**Molecular basis for occlusion of the jeilongvirus receptor-binding site**

**by the elongated C-terminus**

This document includes Supplementary Table S1−S2 and Supplementary Figures S1−S8, Supplementary Figure Legends, and Supplementary References.


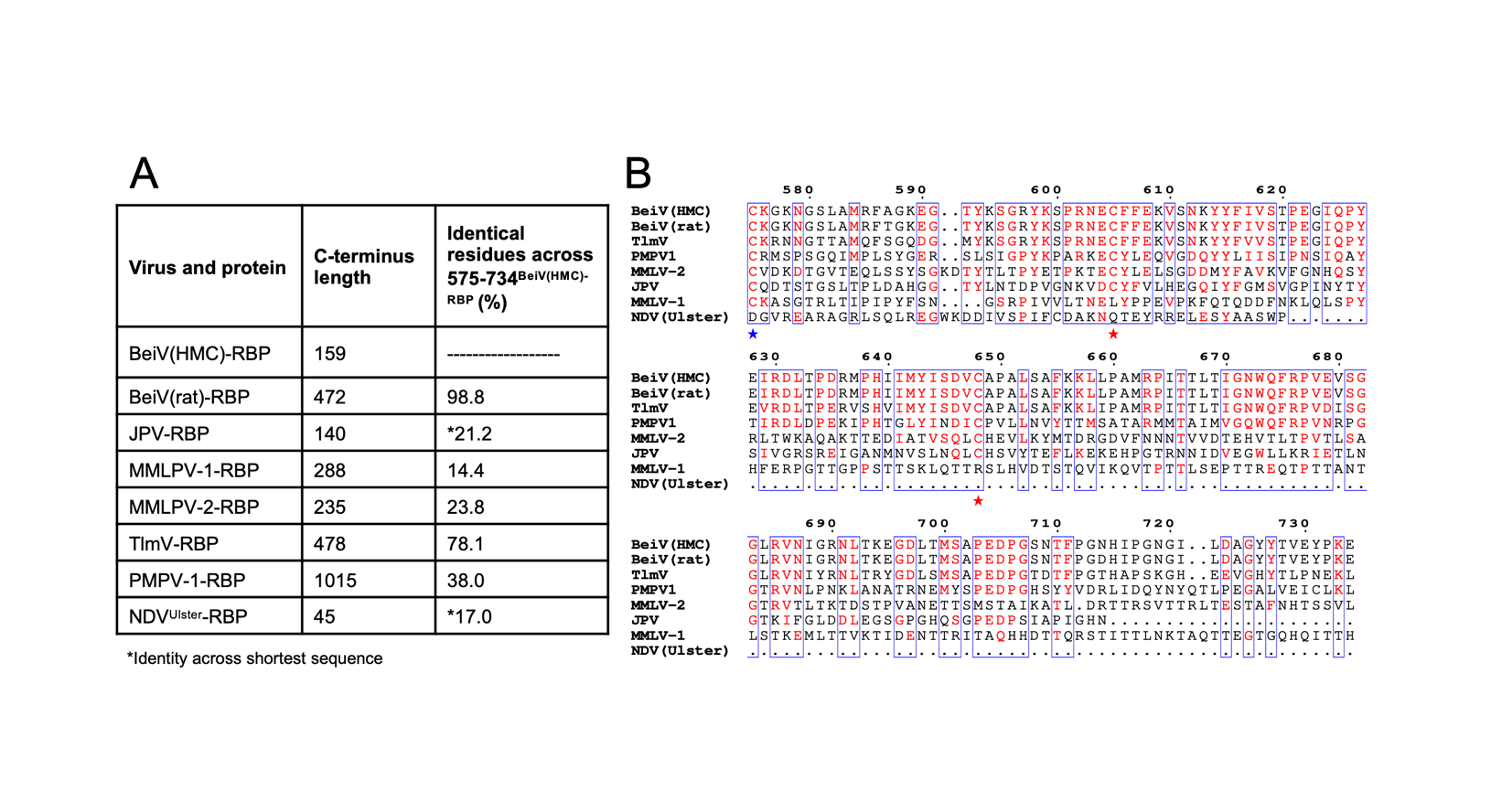


**Figure S1: Sequence analysis of the C-terminal extension found on rodent-borne Jeilongvirus receptor binding proteins (RBPs).** (**A**) A table detailing the length and amino acid conservation of rodent-borne jeilongviral RBP C-terminal extensions. The final column details the percentage conservation between residues K575−Μ734 of the RBP of Beilong virus originating from human kidney mesangial cells (BeiV(HMC)-RBP) with the analogous region in the other jeilongviral RBPs: rat isolated BeiV(rat)-RBP, J paramyxovirus RBP (JPV-RBP), Mount Mabu Lophuromys paramyxovirus 1 and 2 (MMLPV-1/2), Tailam virus (TlmV), Pohorje Myodes paramyxovirus 1 (PMPV-1), and Newcastle Disease virus (NDV) Ulster strain (3-9). The alignment was performed using MultAlin (10), and conservation determined by number of identical residues across the specified region of protein. (**B**) Amino acid sequence alignment of the initial region of the jeilongviral RBP C-terminal extensions, revealing a conserved disulphide bonding pattern (stars). Cys575 (blue star) forms a disulfide bond with Cys163 (not shown), and the residues Cys605−Cys648 (red star) form an intradomain disulfide. Alignment was performed with MultAlin (10) and plotted with ESPRIPT (11).


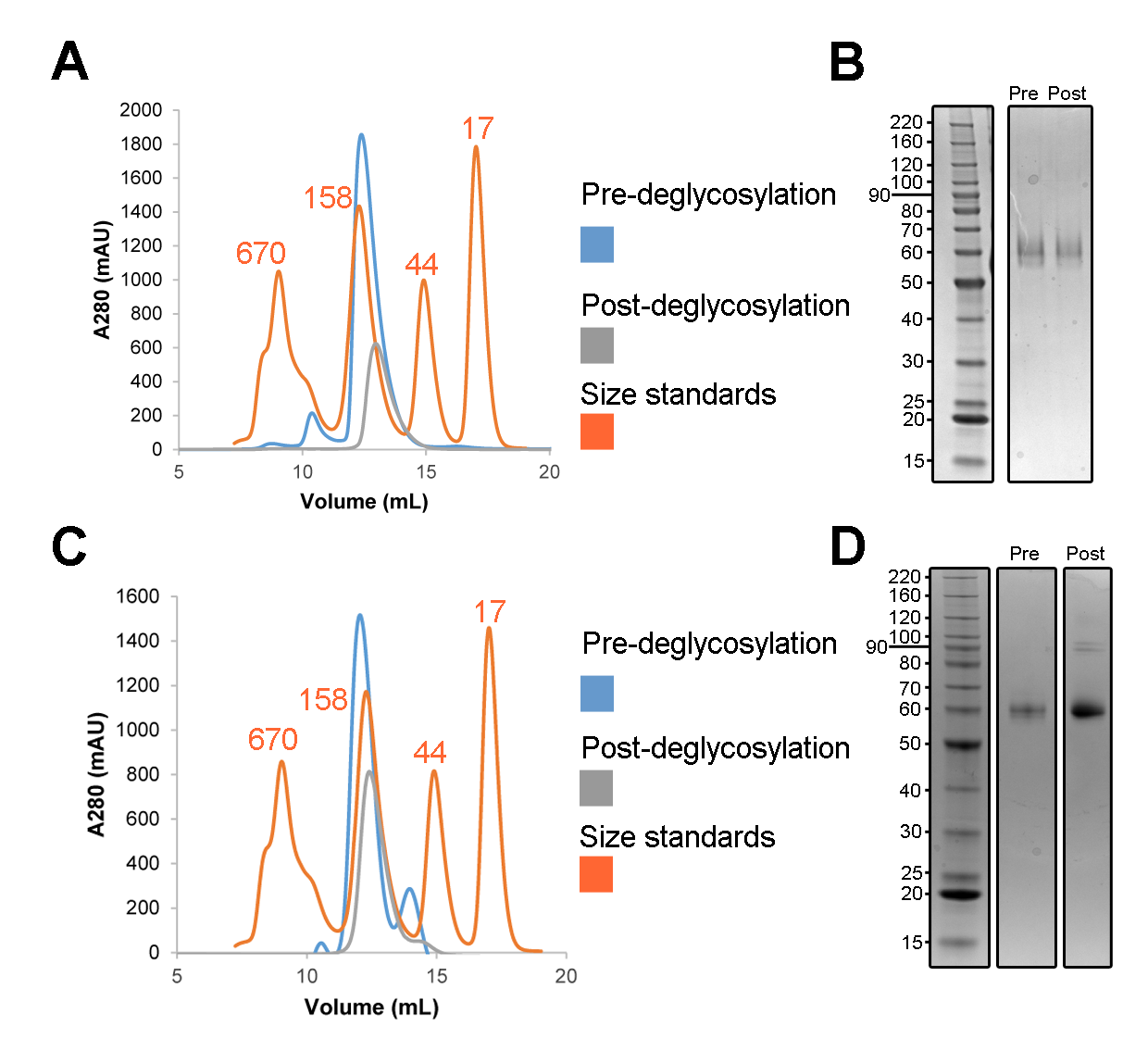


**Figure S2: Purification of JPV-RBP and BeiV(HMC)-RBP.** (**A**) SEC of JPV-RBP_β+_ (Glu149−Asn709) expressed transiently in the presence of kifunensine (13), pre- (blue) and post-deglycosylation (grey) with EndoF1, aligned against gel filtration standards (BioRad, orange). Minor differences in observed elution volume are likely to be more reflective of differences in experimental conditions (*e.g.*, sample volume and concentration) than changes to protein molecular mass, which were not observed by SDS-PAGE. (**B**) The SDS-PAGE analysis of JPV-RBP_β+_ purification pre- and post-deglycosylation does not indicate a shift following treatment with EndoF1. (**C**) SEC of BeiV(HMC)-RBP_β+_ (Asn145−Glu734) expressed stably in the presence of kifunensine, pre- (blue) and post-deglycosylation (grey) with EndoF1, aligned against gel filtration standards (BioRad, orange). (**D**) SDS-PAGE analysis of BeiV-RBP_β+_ pre- and post-deglycosylation reveals no shift following treatment with EndoF1. In both panels B and D, samples were run under reducing conditions, alongside Benchmark™ Protein Ladder (Thermo, left).


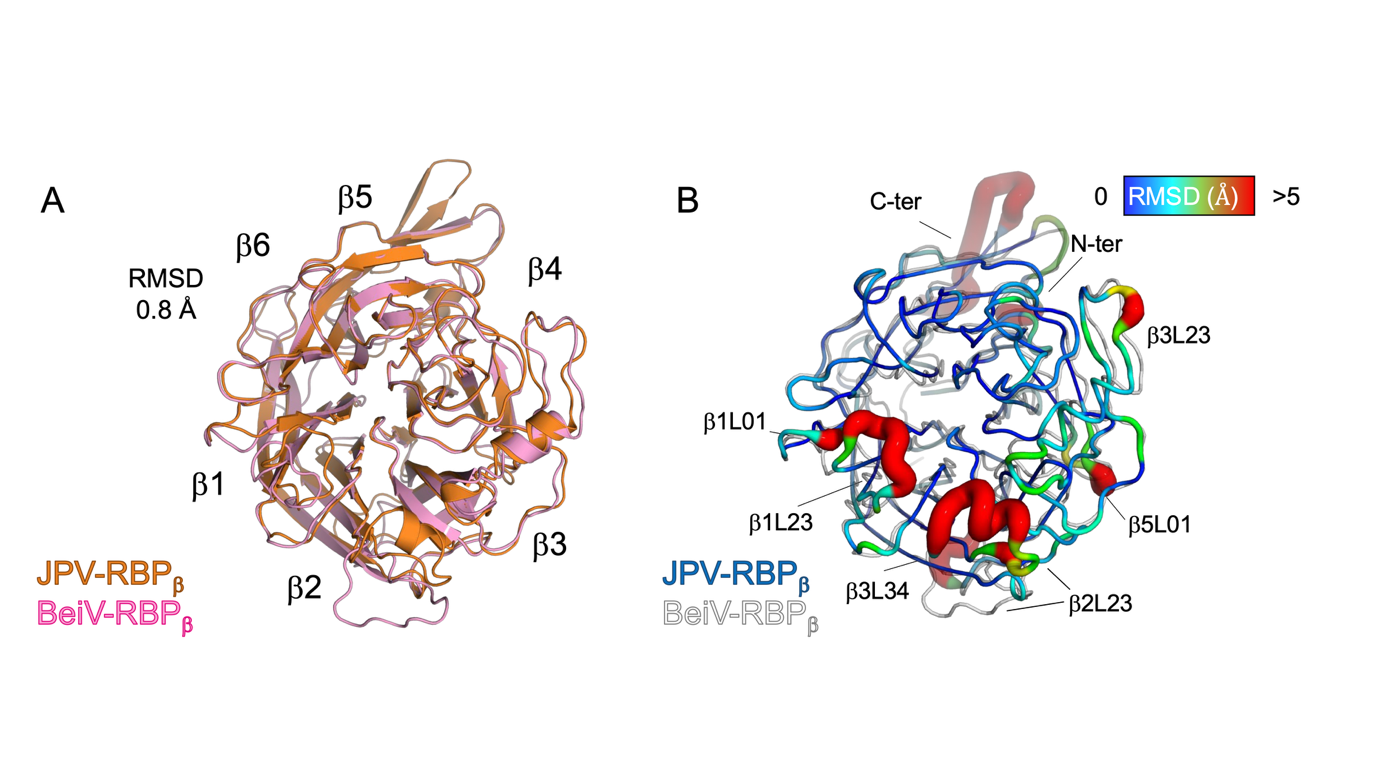


**Figure 3: Similarities between the BeiV and JPV β-propellers.** (**C**) An overlay of the JPV-RBP (orange) and BeiV(HMC)-RBP (pink) β-propellers. (**D**) An overlay JPV-RBP (colored blue to red) and BeiV-RBP (light gray) in cartoon putty. Variation in root-mean-square deviations (RMSD) is represented by a color scale and the thickness of the chain, with blue/thin illustrating the least RMSD and red/thick the largest RMSD between equivalent Cα atoms of the two structures.


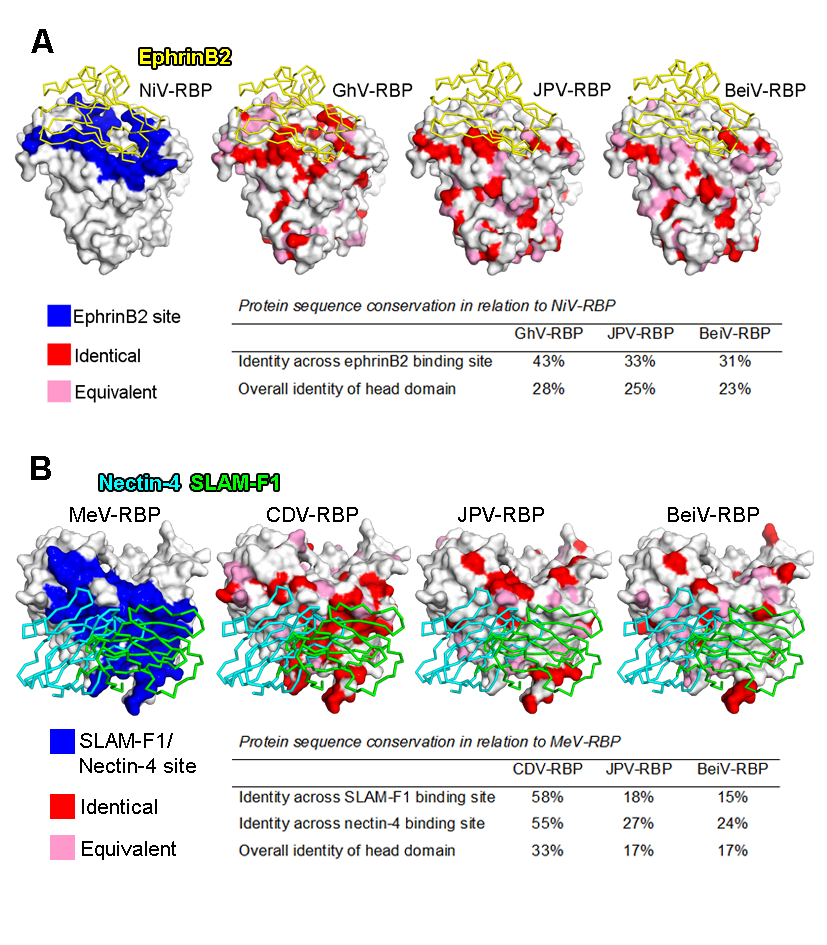


**Figure S4: Mapping of JPV-RBP and BeiV-RBP sequence conservation onto henipaviral and morbilliviral RBPs.** (**A**) NiV-RBP (PDB ID 2VSM) (14)) is shown as a surface with ephrinB2 shown as a yellow ribbon and the cognate receptor-binding footprint colored blue (far left). Sequence conservation of GhV-RBP (second left), JPV-RBP_β_ (second right), and BeiV(HMC)-RBP_β+_ (far right), with NiV-RBP mapped onto the surface of NiV-RBP. Consistent with the known shared usage of ephrinB2, GhV-RBP and NiV-RBP share an elevated level of sequence conservation at the receptor binding site. This feature of elevated sequence conservation was not observed using the same approach with NiV-RBP and the narmoviral RBPs, suggesting that JPV-RBP_β_ and BeiV(HMC)-RBP_β+_ do not utilize ephrin receptors. Residues colored red are identical, those that are pink are equivalent residues. (**B**) MeV-RBP (PDB ID 3ALZ) ((15)) is shown in surface representation with SLAM-F1 (green) and nectin-4 (cyan) shown in ribbon representation and the cognate joint receptor-binding footprint coloured blue (far left). Sequence conservation of CDV-RBP (second left), JPV-RBP_β_ (second right), and BeiV(HMC)-RBP_β+_ (far right), with MeV-RBP is mapped onto the surface of MeV-RBP. Residues colored red are identical, those colored pink are equivalent. Consistent with the shared usage of SLAMF1/nectin-4, MeV-RBP and CDV-RBP share an elevated level of sequence conservation at the receptor binding sites. This feature of elevated sequence conservation was not observed using the same approach with MeV-RBP and the jeilongviral RBPs, suggestive that JPV-RBP_β_ and BeiV(HMC)-RBP_β+_ do not utilize SLAMF1/nectin-4 receptors.


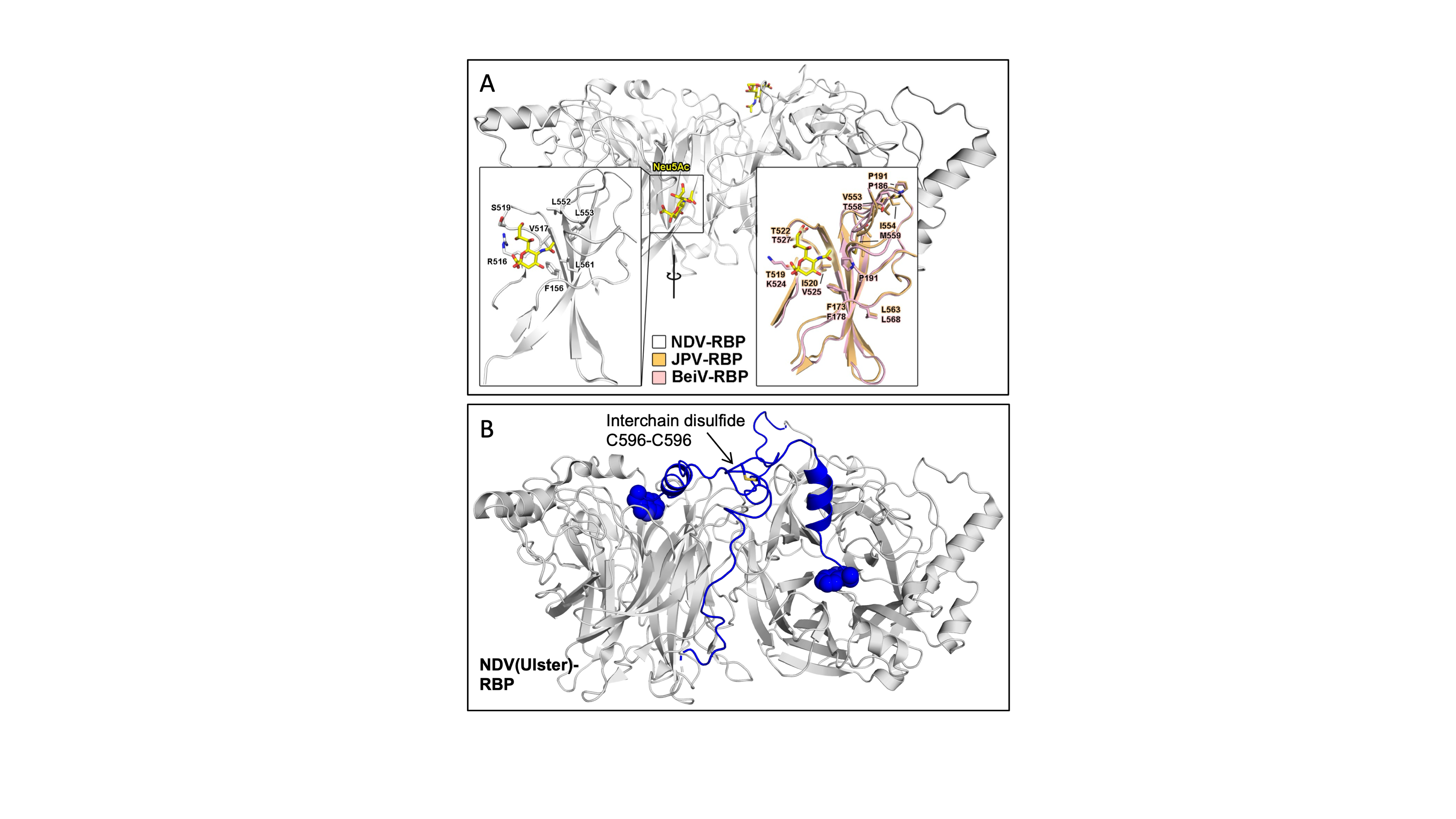


**Figure S5**: **Positioning of the NDV-RBPUlster secondary binding site and interchain disulfide bond**. (**A**) Although JPV-RBP and BeiV(HMC)-RBP did not exhibit observable N-acetylneuraminic acid (Neu5Ac) binding activity, the jeilongvirus RBPs possess a limited level of sequence conservation with residues proximal to the secondary site observed for NDV RBP. The structure of NDV-RBP_Ulster_ is shown as a white cartoon, with the observed Neu5Ac bound ligand shown (PDB ID 1USR) (16). JPV RBP_β_ (orange cartoon) and BeiV(HMC)-RBP_β_ (pink cartoon) are overlayed within a zoom-in panel at the NDV-RBP secondary binding site with the superposed position of Neu5Ac shown. (**B**) The NDV-RBP^Ulster^ dimer (PDB ID 4FZH) shown in cartoon representation with the β-propeller head-domain colored light gray and the C-terminal extension colored blue. The interchain disulfide that connects the C-terminal extensions of the dimer are shown as sticks. The C-terminus of the resolved structure is shown as spheres and colored blue.


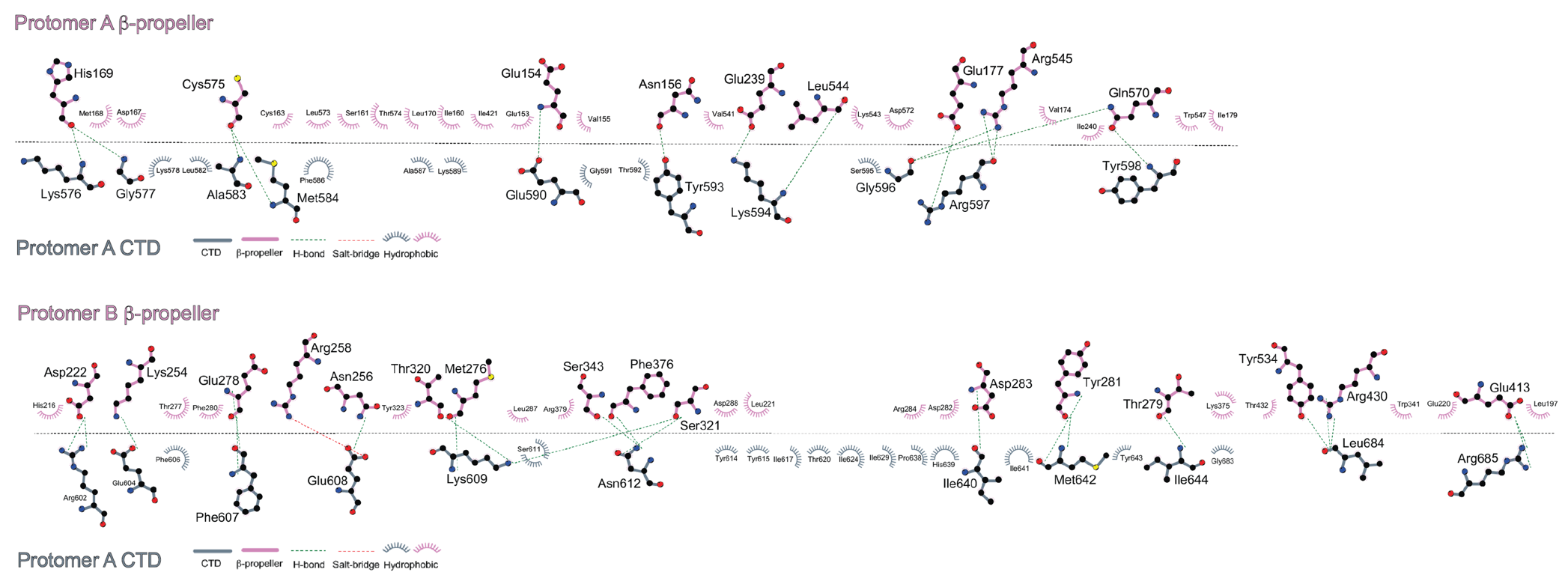


**Figure S6: Dimplot analysis shows the extensive interaction network between the hat-like C-terminal domain (CTD) and protomers A and B of the Bei(HMC)-RBP β-propeller dimer.** CTD and β-propeller residues contributing to interface interactions (salt-bridge, H-bond, hydrophobic) are colored gray and pink, respectively. H-bonds and salt-bridges are shown with dashed lines colored green and red, respectively. Dimplot analysis was performed using the Ligplot+ (v.2.2.8) program (1, 2).


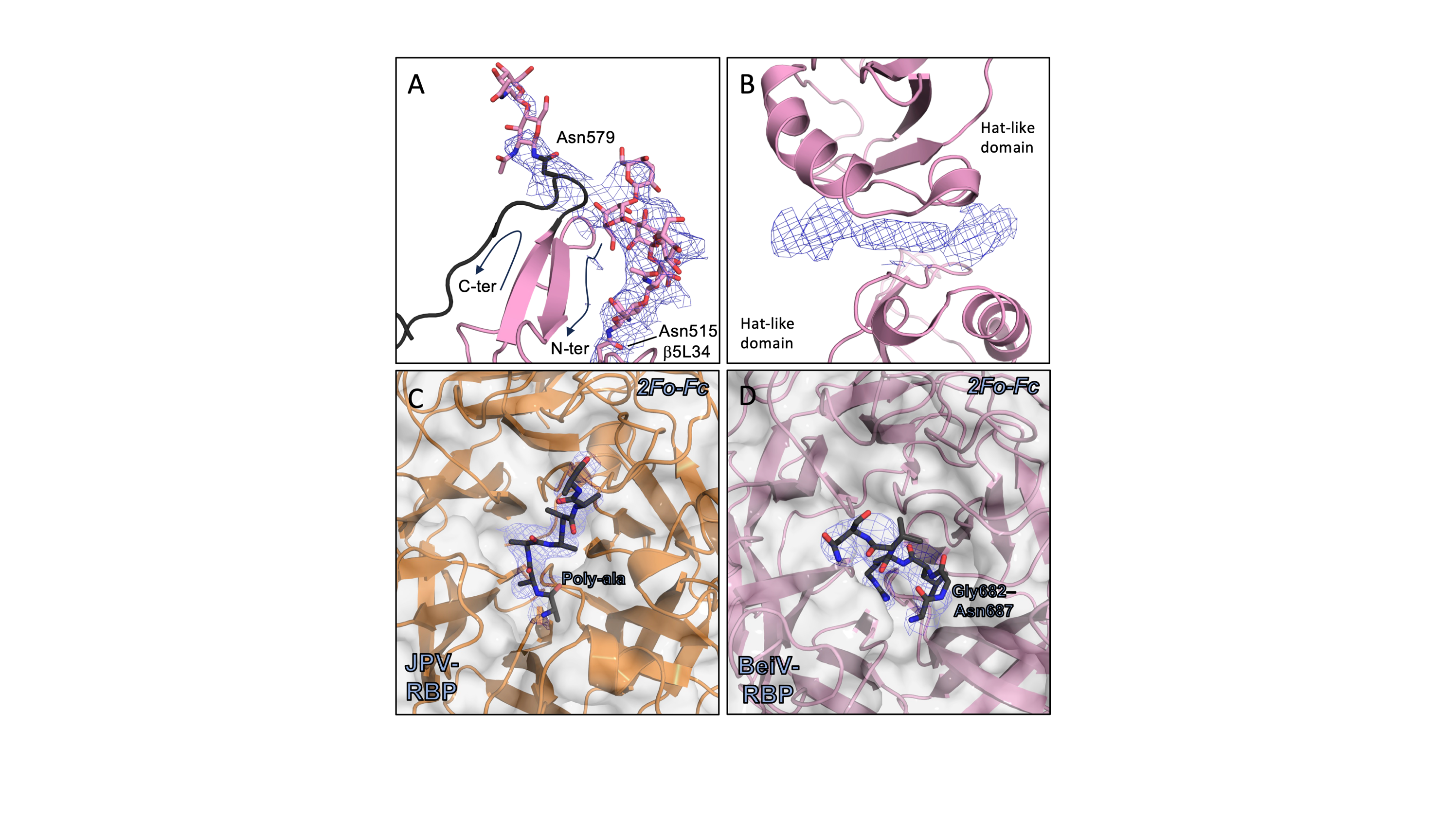


**Figure S7: N-linked glycosylation and unmodelled regions in JPV-RBP and BeiV-RBP structures.** (**A**) BeiV(HMC)-RBPβ+ chain B Asn515 and Asn579 N-linked glycosylation displayed with 2*F*o−*F*c electron density map at sigma level 1.0. The Asn515 N-linked glycan is situated between the loop β5L34, the N-terminus, the beginning of the C-terminal extension and the solvent channel. The Asn579 N-linked glycan is situated at the beginning of the C-terminal extension. (**B**) Further electron density was observed between the hat-like domains between one protomer and its symmetry mate. (**C**) Electron density (sigma level 1.0) treated to one round of refinement with a poly-alanine chain modelled is present in the putative active site of JPV-RBP_β_. Note, as we were unable to assign specific amino acid residues to this density, this polypeptide was not included in the submitted structure. The unassigned electron density (2*F*o−*F*c) within the putative JPV-RBP_β+_ (orange) sialic acid binding site is rendered at 1.0 sigma and colored blue. JPV-RBP_β_ is rendered in cartoon representation. (**D**) For comparison, the electron density in the BeiV(HMC)-RBPβ+ putative active site is shown with modelled residues, Gly682−Asn687.

**
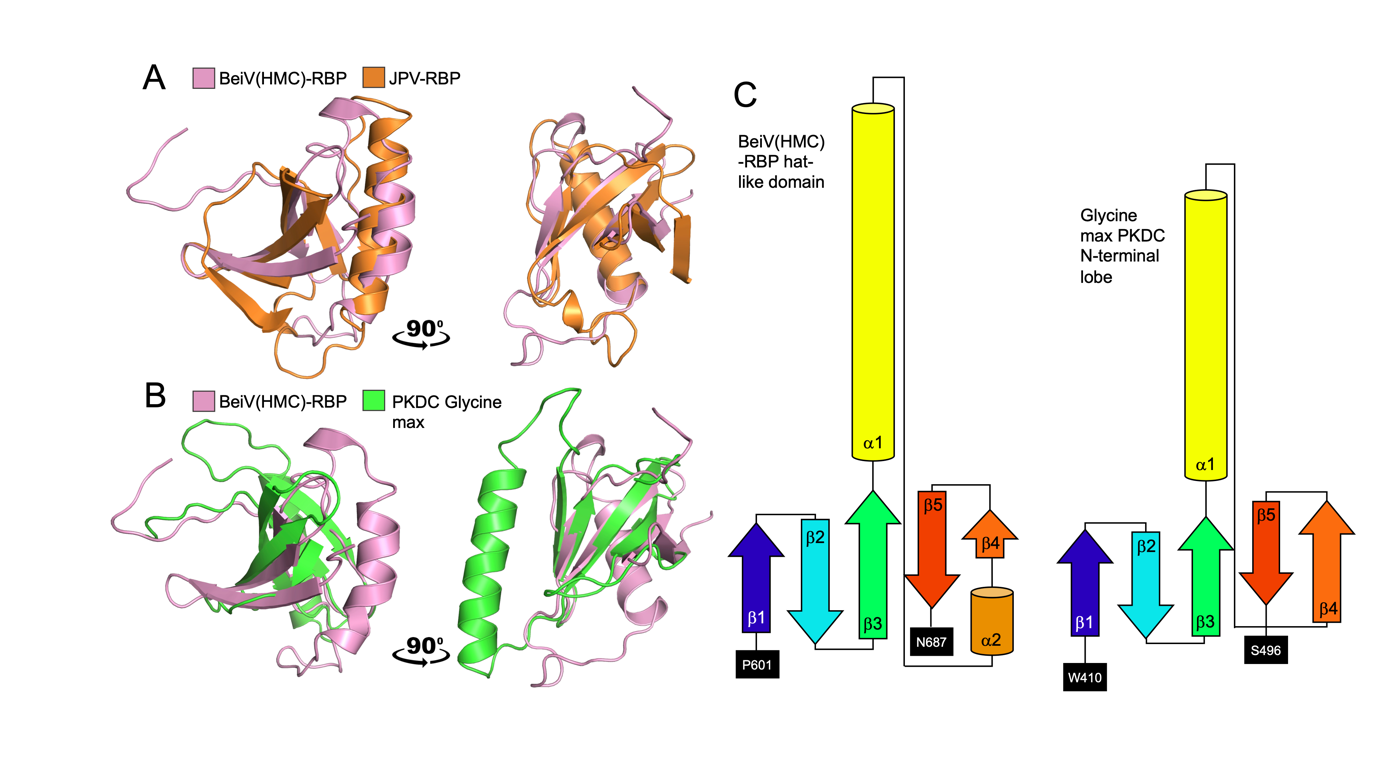
**

**Figure S8: A structural comparison of the hat-like domain of BeiV(HMC)-RBP_β+_ with the structures of closely related proteins**. (**A**) BeiV(HMC)-RBPβ+ hat-like domain (pink) superimposed on the Alphafold 3 (17) predicted structure of the JPV-RBPβ+ hat-like domain (orange). (**B**) BeiV(HMC)-RBPβ+ hat-like domain (pink) superimposed on the Alphafold 3 (17) predicted structure of the N-terminal lobe of the protein kinase domain-containing (PKDC) protein from Glycine max (green). (**C**) The topology of the BeiV(HMC)-RBPβ+ hat-like domain and PKDC protein from Glycine max N-terminal lobe. The β-strands and α-helices are colored according to position from blue to red, N-terminus to C-terminus.

| **Data collection statistics** |  |
| --- | --- |
| Beamline | DLS I04-1 |
| Spacegroup | P 6_(1)_ |
| Cell dimensions |  |
| a, b, c (Å) | 164.2 164.2 112.5 |
| α, β, γ (°) | 90.0 90.0 120.0 |
| Resolution range (Å) | 60.11–2.20  (2.24−2.20)^a^ |
| Wavelength (Å) | 0.9282 |
| R_merge_ | 0.18 (2.34) |
| R_meas_ | 0.19 (2.46) |
| R_pim_ | 0.06 (0.76) |
| I/σ I | 9.6 (1.3) |
| CC_1/2_ | 0.99 (0.48) |
| Completeness (%) | 100 (100) |
| Multiplicity | 10.2 (10.4) |
|  |  |
| **Refinement collection statistics** |  |
| Resolution (Å) | 46.40–2.20  (2.23−2.20) |
| No. reflections | 87,311 |
| Rwork / Rfree^b^ (%) | 18.8/20.6 |
| No. atoms | 7,081 |
| Protein | 6,795 |
| Ligand/Ion | 0 |
| Water | 286 |
| *B*-factors |  |
| Protein | 131.9 |
| Ligand | N/A |
| Water | N/A |
| R.m.s deviations^c^ |  |
| Bond lengths (Å) | 0.005 |
| Bond angles (°) | 0.76 |
| Ramachandran analysis^d^ |  |
| Residues in favoured region (%) | 96.09 |
| Residues in allowed region (%) | 3.91 |

**Supplementary Table S1.** Crystallographic data collection and refinement statistics for JPV-RBP_β+_.

^a^ Numbers in parentheses refer to the relevant outer resolution shell.

^b^ Rfree is calculated as for Rwork, but using only 5% of the data which were separated prior to refinement.

^c^ r.m.s deviations: root mean square deviation from ideal geometry.

^d^ Determined using the Molprobity server (18).

| **Data collection statistics** |  |
| --- | --- |
| Beamline | DLS I24 |
| Spacegroup | C 2 2 2_(1)_ |
| Cell dimensions |  |
| a, b, c (Å) | 88.0 162.4 226.1 |
| α, β, γ (°) | 90.0 90.0 90.0 |
| Resolution range (Å) | 73.18–3.50  (3.56−3.50)^a^ |
| Wavelength (Å) | 0.9686 |
| R_merge_ | 0.14 (0.95) |
| R_meas_ | 0.15 (1.00) |
| R_pim_ | 0.04 (0.29) |
| I/σ I | 13.8 (2.5) |
| CC_1/2_ | 1.00 (0.87) |
| Completeness (%) | 99.8 (99.2) |
| Multiplicity | 12.9 (11.5) |
|  |  |
| **Refinement collection statistics** |  |
| Resolution (Å) | 55.23–3.50  (3.68−3.50) |
| No. reflections | 20,626 |
| Rwork / Rfree^b^ (%) | 24.6/27.3 |
| No. atoms | 8,592 |
| Protein | 8,592 |
| Ligand/Ion | 0 |
| Water | 0 |
| *B*-factors |  |
| Protein | 130.7 |
| Ligand | N/A |
| Water | N/A |
| R.m.s deviations^c^ |  |
| Bond lengths (Å) | 0.002 |
| Bond angles (°) | 0.57 |
| Ramachandran analysis^d^ |  |
| Residues in favoured region (%) | 93.0 |
| Residues in allowed region (%) | 7.0 |

**Supplementary Table S2.** Crystallographic data collection and refinement statistics for BeiV(HMC)-RBP.

^a^ Numbers in parentheses refer to the relevant outer resolution shell.

^b^ Rfree is calculated as for Rwork, but using only 5% of the data which were separated prior to refinement.

^c^ r.m.s deviations: root mean square deviation from ideal geometry.

^d^ Determined using the Molprobity server (18).

**Supplementary References**

1. A. C. Wallace, R. A. Laskowski, J. M. Thornton, LIGPLOT: a program to generate schematic diagrams of protein-ligand interactions. *Protein Eng* **8**, 127-134 (1995).

2. R. A. Laskowski, M. B. Swindells, LigPlot+: multiple ligand-protein interaction diagrams for drug discovery. *J Chem Inf Model* **51**, 2778-2786 (2011).

3. L. Ch'ng *et al.*, Evolution and ecology of Jeilongvirus among wild rodents and shrews in Singapore. *One Health Outlook* **5**, 19 (2023).

4. Y. Zhang *et al.*, Genetic Diversity and Expanded Host Range of J Paramyxovirus Detected in Wild Small Mammals in China. *Viruses* **15** (2022).

5. B. Vanmechelen *et al.*, Discovery and genome characterization of three new Jeilongviruses, a lineage of paramyxoviruses characterized by their unique membrane proteins. *BMC Genomics* **19**, 617 (2018).

6. P. C. Y. Woo *et al.*, Comparative genome and evolutionary analysis of naturally occurring Beilong virus in brown and black rats. *Infect Genet Evol* **45**, 311-319 (2016).

7. H. Schomacker, P. L. Collins, A. C. Schmidt, In silico identification of a putative new paramyxovirus related to the Henipavirus genus. *Virology* **330**, 178-185 (2004).

8. M. H. Jun, N. Karabatsos, R. H. Johnson, A new mouse paramyxovirus (J virus). *Aust J Exp Biol Med Sci* **55**, 645-647 (1977).

9. N. S. Millar, P. Chambers, P. T. Emmerson, Nucleotide sequence of the fusion and haemagglutinin-neuraminidase glycoprotein genes of Newcastle disease virus, strain Ulster: molecular basis for variations in pathogenicity between strains. *J Gen Virol* **69 ( Pt 3)**, 613-620 (1988).

10. F. Corpet, Multiple sequence alignment with hierarchical clustering. *Nucleic Acids Res* **16**, 10881-10890 (1988).

11. X. Robert, P. Gouet, Deciphering key features in protein structures with the new ENDscript server. *Nucleic Acids Res* **42**, W320-324 (2014).

12. Z. Dosztanyi, Prediction of protein disorder based on IUPred. *Protein Sci* **27**, 331-340 (2018).

13. V. T. Chang *et al.*, Glycoprotein structural genomics: solving the glycosylation problem. *Structure* **15**, 267-273 (2007).

14. T. A. Bowden *et al.*, Structural basis of Nipah and Hendra virus attachment to their cell-surface receptor ephrin-B2. *Nat Struct Mol Biol* **15**, 567-572 (2008).

15. T. Hashiguchi *et al.*, Structure of the measles virus hemagglutinin bound to its cellular receptor SLAM. *Nat Struct Mol Biol* **18**, 135-141 (2011).

16. V. Zaitsev *et al.*, Second sialic acid binding site in Newcastle disease virus hemagglutinin-neuraminidase: implications for fusion. *J Virol* **78**, 3733-3741 (2004).

17. J. Abramson *et al.*, Accurate structure prediction of biomolecular interactions with AlphaFold 3. *Nature* **630**, 493-500 (2024).

18. I. W. Davis *et al.*, MolProbity: all-atom contacts and structure validation for proteins and nucleic acids. *Nucleic Acids Res* **35**, W375-383 (2007).
